# Supplementary material for: Numerical study of probe parameters on performance of a transonic axial compressor
Source: PLoS One. 2021 Jan 25;16(1):e0245711. doi: 10.1371/journal.pone.0245711 (PMC7833163; doi:10.1371/journal.pone.0245711)
Supplement: Attachment — Submitted filename: Response to reviewers.pdf [file pone.0245711.s001.pdf]

Comments about submission PONE-D-20-26128

Thankyou for your comments concerning our manuscript entitled “Numerical Study of Probe Parameters on Performance of a Transonic Axial Compressor. Authors have studied all the comments carefully and have made corrections accordingly. Revised portions are highlighted in the revised manuscript with yellow color (i.e., Revised Manuscript with track changes.pdf). The response to each of the reviewer comments is presented below:

Reviewer #1:

1. **how many is the reference pressure in numerical process?**

Response: It is now mentioned in Section 3.1 (Page 5 of 15) as: “Whereas for normalizing, the m_{choke} , T_{ref} and P_{ref} values were 20.93 kg/s, 288K and 101.325kPa, respectively”. (Lines # 123-124)

2. **the keywords need be re-checked carefully. ANSYS-CFX should be deleted.**

Response: Thankyou for the kind suggestion. It is now removed and replaced with “measurement” as the most suitable keyword in our revised-manuscript. (Before Line # 01)

3. **in Introduction, the author should add some references about the non-equilibrium condensation in compressor, such as [DOI: 10.1016/j.applthermaleng.2020.115090], [DOI: 10.1016/j.ijmultiphaseflow.2019.03.005], and so on.**

Response: The introduction now contains one paragraph citing the related references. (i.e., the 2nd last paragraph of Section 1 (on Lines # 40-50), in highlighted manuscript with track changes.

4. **in computational section, the control equations should be presented.**

Response: The equations # 1-5 have now been added to the paper and are highlighted in the marked version with track changes (on Lines # 71-78).

5. **in Figure 1, the figure1(d) need be re-charted to increase resolution.**

Response: The issue has been resolved, and the resolution quality of all the figures in the manuscript has been increased and are now legible.

6. **the resolution of Figure 7 is too low. you can refer the reference [DOI: 10.1016/j.energy.2019.115982], [DOI: 10.1016/j.ijmultiphaseflow.2019.103083].**

Response: The issue has been resolved, and the resolution quality of all the figures in the manuscript has been increased and are now legible.

7. **the figure 6 and 7 should be re-charted, referring the reference [DOI: 10.1016/j.applthermaleng.2019.114388], [DOI: 10.1016/j.enconman.2018.12.001]**

Response: The issue has been resolved, and the resolution quality of all the figures in manuscript has been increased and are now legible.

Reviewer #2:

1. **The resolution for Figures (1d, 5 &6) should be enhanced to at least 300dpi.**

Response: The issue has been resolved, and the resolution quality of all the figures in manuscript has now been increased to 300 dpi and are now legible.

2. **The authors should mention the "Pref" and "Tref" values in text, which they used to plot Fig.1(c,d), and also the mass flow rate value used for normalizing.**

Response: It is now mentioned in Section 3.1 (Page 5 of 15) as: "Whereas for normalizing, the m_{choke} , T_{ref} and P_{ref} values were 20.93 kg/s, 288K and 101.325kPa, respectively". (Lines # 123-124)

3. **The Figures 3&4 which show the characteristics maps for total pressure ratio and efficiency is currently a continuous line which is right but, it should display like "respective points connected with lines" (from choke to near stall).**

Response: Figures 3&4 are now corrected and re-drawn as "points connected with lines," according to the reviewer's suggestion.
